# Effects of Virtual Reality Education on Procedural Pain and Anxiety During Venipuncture in Children: A Randomized Clinical Trial

**DOI:** 10.3389/fmed.2022.849541

**Published:** 2022-04-07

**Authors:** Jung-Hee Ryu, Sung-Hee Han, Sang Mee Hwang, Jiyoun Lee, Sang-Hwan Do, Jin-Hee Kim, Jin-Woo Park

**Affiliations:** ^1^Medical Virtual Reality Research Group, Seoul National University College of Medicine, Seoul, South Korea; ^2^Department of Anesthesiology and Pain Medicine, Seoul National University Bundang Hospital, Seongnam, South Korea; ^3^Department of Anesthesiology and Pain Medicine, Seoul National University College of Medicine, Seoul, South Korea; ^4^Department of Laboratory Medicine, Seoul National University Bundang Hospital, Seongnam, South Korea

**Keywords:** anxiety, children, education, pain, venipuncture, virtual reality

## Abstract

**Background:**

Venipuncture is one of the most frequent and frightening medical procedures for children. This randomized clinical trial aimed to evaluate whether pre-procedural immersive virtual reality (VR) education could decrease pain and anxiety during venipuncture procedure of children.

**Methods:**

Sixty children scheduled for venipuncture at the phlebotomy unit were randomized into either the control or VR group. Before the procedure, children of the control group received conventional simple verbal instructions, whereas those of the VR group experienced a 4-min VR education regarding venipuncture. The primary outcome was the pain and anxiety of pediatric patients assessed with the children’s hospital of eastern ontario pain scale. Secondary outcomes were parental satisfaction, venipuncture time, repeated procedure and procedural difficulty rated by phlebotomists.

**Results:**

The pain and anxiety score during the procedure was significantly lower in the VR group than in the control group (median [IQR], 6.0 [5.0–7.0] vs. 8.0 [6.0–9.8], *P* = 0.001). Parental satisfaction about the procedural process were higher in the VR group than in the control group (*P* = 0.029), and the degree of procedural difficulty was lower in the VR group, compared to the control group (*P* = 0.026).

**Conclusion:**

The preprocedural VR education significantly reduced pain and anxiety of children and decreased the procedural difficulty of phlebotomists during venipuncture procedure.

**Clinical Trial Registration:**

University hospital Medical Information Network Clinical Trials Registry (registration number: UMIN000042968, date of registration: January 9, 2021, URL: https://upload.umin.ac.jp/cgi-open-bin/ctr_e/ctr_view.cgi?recptno=R000049043).

## Introduction

Venipuncture is one of the most frequently performed procedures in children. Approximately 50–80% of children aged <12 years experience high levels of pain and distress during venipuncture (1–3). Behavioral interventions to reduce the pain experienced during this procedure are based on the gate-control theory, which suggests that attention, thoughts, and beliefs influence pain sensation (4, 5). Distraction and education are the two most widely adopted behavioral approaches (6, 7). Diverting the attention of children via video games or animated cartoons during venipuncture significantly decreases needle-related pain (7, 8). Additionally, delivering information about painful procedures and teaching coping skills have been reported to alleviate procedural pain and anxiety in children (6, 9).

Virtual reality (VR) refers to a computer-generated realistic environment providing immersive and vivid experiences (10). Recently, VR systems have been utilized to alleviate pain or anxiety during medical procedures (1, 6, 7, 11–16). Distraction using a VR game was reported to effectively reduce “Worst pain” and “Pain unpleasantness” during venipuncture in children (1). Furthermore, VR systems providing procedural information through a simulated experience have been proven as an effective education platform to minimize peri-procedural anxiety in children (13–16). However, these previous studies used VR solely as a distraction tool to manage needle-related pain and anxiety.

To the best of our knowledge, no data exist regarding the effects of VR education on pain and anxiety during venipuncture in children. We hypothesized that VR education could decrease pain and anxiety during venipuncture in children. This randomized clinical trial was aimed to evaluate the effects of immersive VR education about venipuncture on procedural pain and anxiety in children. Additionally, parental satisfaction and procedural outcomes such as venipuncture time, repeated procedure, and difficulty score were also evaluated.

## Materials and Methods

### Study

This prospective randomized clinical trial was approved by the institutional review board of Seoul National University Bundang Hospital (IRB number: B-1911-574-301; date of approval: October 25, 2019) and registered at University hospital Medical Information Network Clinical Trials Registry (registration number: UMIN000042968; date of registration: January 9, 2021). Written informed consent was collected from the parents or guardians of children. Additionally, children aged ≥7 years received detailed instructions on this protocol and signed additional agreements. This study was conducted from February 2, 2021, to June 30, 2021, at Seoul National University Bundang Hospital (SNUBH).

### Patients

Children aged 4–8 years who were scheduled to undergo venipuncture at the phlebotomy unit in SNUBH were enrolled in this study. Children with congenital disorders, hearing or visual impairments, intellectual developmental disabilities, cognitive deficits, epilepsy or seizure history, psychoactive medication prescriptions, and prior experience of venipuncture during the past year were excluded.

### Randomization and Intervention

Enrolled children were randomly allocated to either the control or VR group via a computer-generated randomization code (Random Allocation Software, version 1.0; Isfahan University of Medical Sciences) in a 1:1 ratio. Randomization was performed by an independent researcher who was only in charge of patient assignment, 10 min before venipuncture. An opaque envelope with a randomization number was transferred to another researcher who performed the intervention in a separated area 5 min before entering the phlebotomy unit. Children in the control group received conventional, simple verbal instructions about the procedure, and those in the VR group underwent a 4-min VR education regarding venipuncture. Before the conventional instructions or VR education, the children were asked to indicate the expected venipuncture-related pain on a visual analog scale [range, 0 (no pain) to 10 (the worst pain)]. During the protocol, the children and their parents or guardians were not blinded to the intervention, whereas the evaluator and phlebotomy technicians were blinded to the group allocation.

### Virtual Reality Education

**FIGURE 1:**
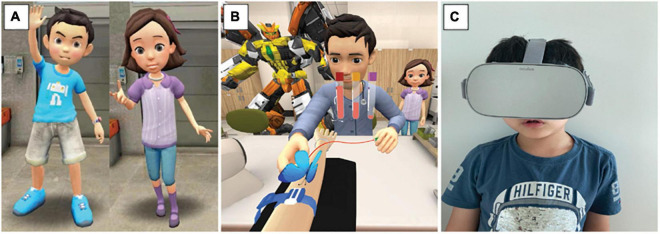
Virtual reality education system. **(A)** Children select Chatan (left) or Mona (right) as a main character; **(B)** and experience venipuncture procedure in a 360° and 3-dimensional virtual phlebotomy unit; **(C)** with a head-mounted virtual reality display.

The VR content was produced in collaboration with a VR producing company (JSC Games, Seoul, South Korea). All equipment and facilities in the phlebotomy unit in SNUBH were measured and rendered three-dimensionally to create a 360° three-dimensional virtual environment where users can experience immersive virtual education from a first-person perspective. The storyline of the VR education was written by phlebotomy technicians and anesthesiologists (J-WP, S-HH) and revised by pediatric psychiatrists in SNUBH. The VR education began with characters of “Hello Carbot” (Choi-Rock Contents Factory, Seoul, South Korea), a famous animated film in the Republic of Korea, greeting the children in front of the phlebotomy unit. The children then selected a main character conducting the education (Chatan or Mona; Figure 1A) according to their preferences. In a friendly tone, Chatan or Mona explained the purpose and process of venipuncture in detail, reminding the children to be brave and to not move during the procedure. The children underwent venipuncture in the VR phlebotomy unit after learning how to position themselves at phlebotomy desk and were encouraged to cooperate appropriately while trying not to feel anxious (Figure 1B). Through a licensing agreement with ChoiRock Contents Factory, we obtained the permission to use the characters. The VR experience was provided with a head-mounted VR display, OculusGo (OculusVR, Menlo Park, CA, United States; Figure 1C).

### Study Outcomes

After the intervention, the children entered the phlebotomy unit and underwent venipuncture as is usually practiced in SNUBH. The primary outcome was children’s anxiety and pain assessed using the Children’s Hospital of Eastern Ontario Pain Scale (CHEOPS), which was measured by a single blinded assessor during the procedure. The CHEOPS assesses six behaviors—cry, facial expression, verbal response, torso, hands, and legs (Supplementary Table 1); each behavior is coded with scores based on its intensity, and the total sum of the scores is considered to assess procedural pain and anxiety (the primary outcome of this study; score range: 4–13) (17).

Secondary outcomes were parental satisfaction and procedural outcomes. After the procedure, the parents or guardians of the children were asked to grade their satisfaction regarding the overall process of venipuncture using a numerical rating scale [range, 0 (very dissatisfied) to 10 (very satisfied)]. The time required for the venipuncture procedure (time from sitting at the phlebotomy desk to successful needle insertion for blood sampling) and the requirement of needle re-insertion due to bad patient cooperation were recorded by the blinded evaluator. Phlebotomy technicians rated the level of difficulty in performing venipuncture using the numerical rating scale [range, 0 (very easy) to 10 (very difficult)] immediately after the patients left the phlebotomy unit.

### Statistical Analysis

Baseline characteristics included age, gender, height, weight, and preprocedural pain expectation. Continuous data are presented as median [interquartile range (IQR)], and categorical variables are presented as a number (percentage). The Mann–Whitney U test was used to compare continuous outcomes between the two groups. Categorical outcomes were analyzed using Fisher’s exact test. We used SPSS, version 21.0 (SPSS Inc., IBM, Chicago, IL, United States) for all statistical analyses. A two-sided *P*-value < 0.05 was considered to be statistically significant.

### Sample Size

We conducted a power analysis using G*Power software, version 3.1.2 (Heinrich Heine University). A previous study reported that the mean and standard deviation of the CHEOPS score during venipuncture in the children were 9.3 and 2.4, respectively (18). A relative reduction in the pain and anxiety scores by 25% during venipuncture was considered clinically significant with respect to the VR education. A sample size of 30 children per group was calculated with a power of 0.9, significance level of 0.05, and an assumed dropout rate of 20%.

## Results

**FIGURE 2:**
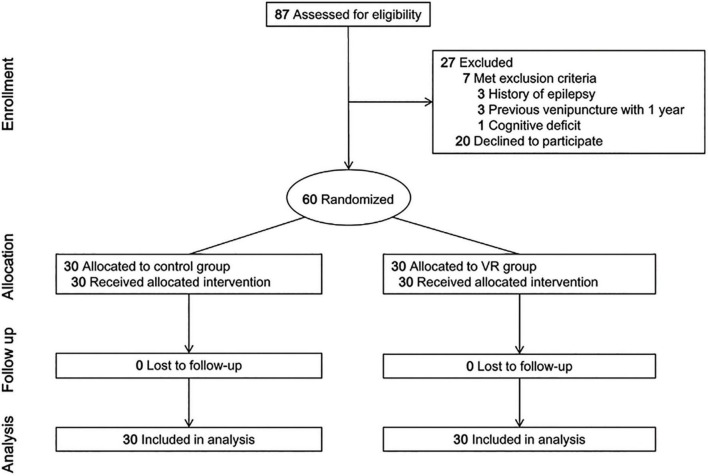
CONSORT flow diagram. VR, virtual reality.

Eighty-seven children undergoing venipuncture were screened, and 27 of them were excluded (seven children met the exclusion criteria and 20 declined to participate). A total of 60 children (control group, 30; VR group, 30) participated in the study and none of them dropped out (Figure 2). The median (IQR) age was 6.0 (5.0–7.0) years in both groups (Table 1). Males accounted for 60.0 and 66.7% of the participants in the control group and the VR group, respectively. Children’s height, weight, and expectation of procedural pain were also comparable between the groups (Table 1).

**TABLE 1:** Patients’ characteristics.

	Control group (*n* = 30)	VR group (*n* = 30)
Age, years	6.0 (5.0-7.0)	6.0 (5.0-7.0)
Male, No. (%)	18 (60.0)	20 (66.7)
Weight, median (IQR), kg	22.0 (18.3-24.8)	21.5 (18.2-24.0)
Height, median (IQR), cm	116.0 (112.0-120.0)	116.5 (110.0-121.5)
Preprocedural pain expectation, median (IQR), VAS	4.0 (0.5-6.0)	4.0 (0.0-4.0)

*IQR, interquartile range; VR, virtual reality; VAS, visual analogue scale.*

Procedural anxiety and pain were assessed via the CHEOPS including six behaviors; the Mann–Whitney U test showed that those were less severe in the VR group. The CHEOPS score during the procedure was significantly lower in the VR group (median [IQR], 6.0 [5.0–7.0]) than in the control group (median [IQR], 8.0 [6.0–9.8]) (*P* = 0.001; Table 2). Parental satisfaction regarding the overall venipuncture process was higher in the VR group (median [IQR], 10.0 [9.0–10.0]) than in the control group (median [IQR], 8.0 [7.3–10.0]) (*P* = 0.029; Table 2).

**TABLE 2:** CHEOPS and parental satisfaction during venipuncture procedure.

	Control group (*n* = 30)	VR group (*n* = 30)	*P* value
CHEOPS, median (IQR)	8.0 (6.0–9.8)	6.0 (5.0–7.0)	0.001
Parental satisfaction score, median (IQR), NRS	8.0 (7.3–10.0)	10.0 (9.0–10.0)	0.029

*CHEOPS, Children’s Hospital of Eastern Ontario Pain Scale; IQR, interquartile range; NRS, numeric rating scale; VR, virtual reality.*

Venipuncture duration was not significantly different between the groups (Table 3). Also, there was no difference in the incidence of repeated procedure. During the protocol, only one child required needle re-insertion due to inappropriate cooperation (in the control group). The degree of procedural difficulty graded by the phlebotomy technicians was lower in the VR group (median [IQR], 1.0 [0.0–2.0]) than in the control group (median [IQR], 2.0 [0.3–5.0]) (*P* = 0.026).

**TABLE 3:** Venipuncture time, the requirement of needle re-insertion, and difficulty score during the procedure.

	Control group (*n* = 30)	VR group (*n* = 30)	*P* value
Venipuncture time, median (IQR), s	50.5 (42.8–72.0)	51.5 (36.0–66.8)	0.956
Repeated procedures, No. (%)	1 (3.3)	0 (0.0)	1.000
Difficulty score for the procedure, median (IQR), NRS	2.0 (0.3–5.0)	1.0 (0.0–2.0)	0.026

*IQR, interquartile range; NRS, numeric rating scale; VR, virtual reality.*

## Discussion

This was the first randomized controlled trial investigating the effects of VR education on pain and anxiety during venipuncture in children, one of the most common and fear-inducing medical procedures in children. VR education 5 min before entering the phlebotomy unit reduced procedural pain and anxiety in children and increased parental satisfaction, compared with conventional simple verbal instructions regarding the venipuncture procedure. Additionally, phlebotomy technicians reported greater ease in performing the procedure with children who had received VR education than with those who were given conventional instructions.

Previous studies have reported that VR education reduced periprocedural or perioperative anxiety in children, which is consistent with our results (13–16). Compared to 2-dimensional video education, 360°C 3-dimensional VR experience of the same content significantly reduced periprocedural anxiety in children undergoing chest radiography, which demonstrated the superior educational effect of VR (14). In the field of psychiatry, VR is utilized as an emerging method of exposure (19, 20). VR exposure in cognitive behavior therapy could be more effective than conventional *in vivo* approach in patients with social anxiety disorders (21). In a recent study, a VR adaptation of the Trier Social Stress Test showed the potential to induce endocrine responses comparable to those observed in the *in vivo* test (22). Clearly, VR technology is evolving into creating immersive and vivid experiences as realistic as *in vivo* experiences (20–23).

Prior to the present study, no data were available regarding the effects of pre-procedural VR education on pain and anxiety in children undergoing painful medical procedures. Immersive education regarding the procedure and a vivid virtual experience using a VR system might modulate the children’s knowledge, attitude, and expectations regarding venipuncture positively; this can influence pain sensation according to the gate-control theory (4, 5). Before the intervention, no significant difference was noted between the two groups in the expectations for needle-related pain.

Unmanaged pain and anxiety associated with medical procedures might not only cause short-term suffering but also lead to negative long-term complications such as post-traumatic stress syndrome, avoidance of medical treatment, and needle phobia (6, 11, 13). Therefore, the fact that VR education significantly decreases pain and anxiety during painful medical procedures is of great clinical value. To measure pain and anxiety objectively in the present study, we utilized the CHEOPS score, which has been applied to evaluate procedural pain and anxiety in children during acute pain-inducing situations such as venipuncture, fracture reduction, burn dressing, and laceration repair (17, 24, 25).

Patient or parental satisfaction is an important indicator of the quality of health care (26–28). Adequate satisfaction improves compliance with treatment; parents who are satisfied with the medical treatment for their child might pay more attention to their child’s health care and carefully follow doctors’ recommendations (29). Appropriate pain management is considered the most critical factor for parental satisfaction (30). Furthermore, venipuncture in children is a fairly challenging and stressful task for phlebotomists (31, 32). When performing needle-related procedures with anxious children (or parents), they report experiencing a high level of stress (31). Because children are easily influenced by the mood or stress of parents and healthcare providers, parental dissatisfaction or difficulty regarding the venipuncture procedure might worsen pain and anxiety in children (4, 31). In this study, encouragingly, the VR group children showed higher parental satisfaction and lower procedural difficulty than the control group children.

At the phlebotomy unit in SNUBH, children showing moderate or severe anxiety are allowed to sit on the parent’s lap in order to allow the parents to easily immobilize the children’s extremities during venipuncture (33). No significant difference was noted in the venipuncture duration and incidence of repeated procedure between the two groups probably because of active parental participation. The position was also reported to reduce children’s fear regarding the procedure (33). However, obvious difference was noted in the behaviors objectively assessed using the CHEOPS between the two groups in this study.

## Conclusion

Immersive and vivid VR education prior to venipuncture significantly decreased procedural pain and anxiety in children, indicating that pre-procedural VR education could be an effective behavioral intervention. Further studies involving pre-procedural VR education in children undergoing painful medical procedures are required to support our findings.

## Data Availability Statement

The original contributions presented in the study are included in the article/Supplementary Material, further inquiries can be directed to the corresponding author.

## Ethics Statement

The studies involving human participants were reviewed and approved by the Institutional Review Board of Seoul National University Bundang Hospital. Written informed consent to participate in this study was provided by the participants’ legal guardian/next of kin. Written informed consent was obtained from the minor(s)’ legal guardian/next of kin, for the publication of any potentially identifiable images included in this article.

## Author Contributions

J-HR and S-HH conceptualized and designed the study, collected data, conducted the initial analysis, and drafted the initial manuscript. SH and JL designed the study, coordinated and supervised data collection, and reviewed the manuscript. S-HD and J-HK designed the study, conducted the initial analysis, and reviewed the manuscript. J-WP conceptualized and designed the study, supervised data collection, conducted the initial analysis, drafted the initial manuscript, and reviewed and revised the manuscript. All authors approved the final manuscript as submitted and agreed to be accountable for all aspects of the work.

## Conflict of Interest

S-HH and J-WP are the co-inventors of the patent, “Medical experience in hospitals provided with VR or AR system”, application of which is pending. The remaining authors declare that the research was conducted in the absence of any commercial or financial relationships that could be construed as a potential conflict of interest.

## Publisher’s Note

All claims expressed in this article are solely those of the authors and do not necessarily represent those of their affiliated organizations, or those of the publisher, the editors and the reviewers. Any product that may be evaluated in this article, or claim that may be made by its manufacturer, is not guaranteed or endorsed by the publisher.
