# Supplementary material for: Effects of Virtual Reality Education on Procedural Pain and Anxiety During Venipuncture in Children: A Randomized Clinical Trial
Source: Front Med (Lausanne). 2022 Apr 7;9:849541. doi: 10.3389/fmed.2022.849541 (PMC9022029; doi:10.3389/fmed.2022.849541)
Supplement: Supplementary file 1 [file Table_1.DOCX]

**Supplementary table 1** Children’s hospital of eastern ontario pain scale (CHEOPS)

| **Criteria** | | **Score** |
| --- | --- | --- |
| **Cry** | No cry | 1 |
|  | Moaning/Crying | 2 |
|  | Screaming | 3 |
| **Facial expression** | Smiling | 0 |
|  | Composed | 1 |
|  | Grimace | 2 |
| **Verbal** | Positive | 0 |
|  | None/Other complaints | 1 |
|  | Pain complaints | 2 |
| **Torso** | Neutral | 1 |
|  | Shifting/Tense/Shivering/Upright/Restrained | 2 |
| **Touch** | Not touching | 1 |
|  | Reach/Touch/Grab/Restrained | 2 |
| **Legs** | Neutral | 1 |
|  | Squirming/Kicking/Drawn Up/Tensed/Standing/Restrained | 2 |
